# Primary health care organization in municipalities of São Paulo, Brazil: a model of care aligned with the Brazilian Unified National Health System’s guidelines

**DOI:** 10.1590/0102-311XEN099723

**Published:** 2024-02-26

**Authors:** Elen Rose Lodeiro Castanheira, Lígia Schiavon Duarte, Mônica Martins de Oliveira Viana, Luceime Olívia Nunes, Thais Fernanda Tortorelli Zarili, Carolina Siqueira Mendonça, Patricia Rodrigues Sanine

**Affiliations:** 1 Faculdade de Medicina de Botucatu, Universidade Estadual Paulista Júlio de Mesquita Filho, Botucatu, Brasil.; 2 Instituto de Saúde, Secretaria de Estado da Saúde de São Paulo, São Paulo, Brasil.; 3 Centro de Ciências Biológicas e da Saúde, Universidade Estadual do Oeste do Paraná, Cascavel, Brasil.

**Keywords:** Primary Health Care, Healthcare Models, Health Services Evaluation, Organization and Administration, Atenção Primária à Saúde, Modelos de Assistência à Saúde, Avaliação de Serviços de Saúde, Organização e Administração, Atención Primaria de Salud, Modelos de Atención de Salud, Evaluación de Servicios de Salud, Organización y Administración

## Abstract

This study analyzes the main organization patterns used by primary health care (PHC) services in municipal networks and evaluates them according to indicators of local management-administration interface. Evaluative research analyzed 461 municipalities in São Paulo, Brazil, that participated in the *Primary Care Services Quality Assessment Survey* (QualiAB) in 2017/2018, classified according to the organizational arrangements composition of 2,472 PHC services. Eight indicators of local management and administration were selected to evaluate the identified patterns. Results indicate two groups of municipalities: homogeneous, with services presenting the same arrangement (43.6%); and heterogeneous, with different arrangements (56.4%). These were subdivided into seven patterns that ranged from homogeneous-traditional, homogeneous-Family Health Strategy, homogeneous-mixed, and different combinations in the heterogeneous group. All indicators showed significant differences between groups (p < 0.001), especially the homogeneous-traditional group, which presented an organizational pattern far from the desired model of a comprehensive and problem-solving PHC. Those integrated with family health units (FHU) and basic health units with community health workers and/or family health teams (BHU/FHU) showed a pattern closer to a comprehensive model - with planning and evaluation actions committed to the local reality and qualification of care. Implementation of federal and state policies are essential for defining the PHC health care model adopted by municipalities.

## Resumo

Este trabalho tem como objetivo analisar os principais padrões de organização das redes municipais de serviços de atenção primária à saúde (APS) e avaliá-los segundo os indicadores de interface entre gestão e gerenciamento local. Trata-se de pesquisa avaliativa que analisou 461 municípios de São Paulo, Brasil, que participaram do *Inquérito de Avaliação da Qualidade de Serviços de Atenção Básica* (QualiAB) em 2017/2018, classificados segundo a composição dos arranjos organizacionais de 2.472 serviços de APS. Para avaliar os padrões identificados, foram selecionados oito indicadores de gestão e gerenciamento local. Os resultados apontam dois grupos de municípios: homogêneos, com serviços de um mesmo arranjo (43,6%); e heterogêneos, com diferentes arranjos (56,4%). Os grupos foram subdivididos em sete padrões que variaram entre homogêneo-tradicional, homogêneo-Estratégia Saúde da Família, homogêneo-misto e diferentes combinações no grupo heterogêneo. Todos os indicadores apontaram diferenças significativas entre os grupos (p < 0,001), com destaque para o grupo homogêneo-tradicional, com padrão organizacional distante do modelo desejado para uma APS abrangente e resolutiva, enquanto aqueles com unidades de saúde da família (USF), e com unidades básicas com agentes comunitários de saúde e/ou equipes de saúde da família (UBS/USF) demonstraram um padrão mais aproximado desse modelo - com ações de planejamento e avaliação comprometidos com a realidade local e com a qualificação do trabalho. Discute-se a importância das políticas implementadas pela gestão federal e estadual e seu poder de indução na definição do modelo de atenção à saúde na APS dos municípios.

## Resumen

El trabajo tiene el objetivo de analizar los principales patrones de organización de las redes municipales de servicios de atención primaria de salud (APS) y evaluarlos conforme los indicadores de interfaz entre la dirección y gestión local. Se trata de una investigación evaluativa que analizó 461 municipios de São Paulo, Brasil, que participaron de la *Encuesta de Evaluación de la Calidad de los Servicios de Atención Primaria* (QualiAB) en 2017/2018, clasificados según la composición de los arreglos organizativos de 2.472 servicios de APS. Para evaluar los patrones identificados, se seleccionaron ocho indicadores de dirección y gestión local. Los resultados indican dos grupos de municipios: homogéneos, con servicios de un mismo arreglo (43,6%) e heterogéneos, con arreglos diferentes (56,4%). Los grupos se subdividieron en siete patrones que iban desde homogéneo-tradicional, homogéneo-Estrategia de Salud de la Familia, homogéneo-mixto y diferentes combinaciones en el grupo heterogéneo. Todos los indicadores señalaron diferencias significativas entre los grupos (p < 0,001), con destaque para el grupo homogéneo-tradicional, con patrón organizativo alejado del modelo deseado para una APS completa y resolutiva, mientras aquellos con unidades de salud de la familia (USF), y con unidades básicas con agentes comunitarios de salud y/o equipos de salud de la familia (UBS/USF) demostraron un patrón más cercano a este modelo -con acciones de planificación y evaluación comprometidas con la realidad local y con la calificación del trabajo. Se discute la importancia de las políticas implementadas por la gestión federal y la gestión estatal y su poder de inducción para definir el modelo de atención a la salud en la APS de los municipios.

## Introduction

In Brazil, structuring primary health care (PHC) services, especially when organized according to the Family Health Strategy (FHS) model, is essential for consolidating the principles and guidelines put forward by the Brazilian Unified National Health System (SUS) and for obtaining better results in favor of more equitable, comprehensive, and universal care ^1^ ^,^ ^2^.

But this FHS-based consolidation took place in a heterogenous manner and has been further compromised by political instabilities and setbacks over the past few years. Weakened PHC valuation policies, aggravated by the COVID-19 pandemic, has also been reported internationally ^3^. In countries where, as in Brazil, systems in which PHC plays a central role in the network organization are structured such as England, Canada and France, the crisis scenario caused by economic liberalism associated with fiscal austerity is reiterated, which leaves this level of care precarious, compromising community and comprehensive care, as well as the health system as a whole ^3^ ^,^ ^4^ ^,^ ^5^ ^,^ ^6^ ^,^ ^7^.

This international scenario finds echo in Brazil following the changes implemented as of 2017. National transition from chronic underfunding to defunding and the implementation of measures favoring privatization and social targeting - which prioritizes care for those who lack access to private health plans and insurance ^4^ ^,^ ^8^ ^,^ ^9^ - are examples of policy propositions that stress the private biomedical model. Approval of the Previne Brasil program denounces this movement, which puts the universal right to health at risk under the fallacy of the inefficiency of public management ^3^ ^,^ ^4^ ^,^ ^5^ ^,^ ^6^ ^,^ ^7^ ^,^ ^10^ ^,^ ^11^ ^,^ ^12^.

In this scenario, besides changes in the PHC financing and qualification policies by the federal and state management, municipal managers play a strategic role as key agents in guiding practices and incorporating guidelines into the services of their territories, as they are directly responsible for organizing the municipal PHC services network, including management of the available resources and definition of the care model ^13^ ^,^ ^14^ ^,^ ^15^ ^,^ ^16^.

In a context of repeated opposition and dispute around health projects, but also of an expected resumption of policies for SUS strengthening, analyzing the different PHC organizational arrangements in the period that preceded such political changes provides elements to better understand the present and the measures necessary to effect PHC as an organizer of care and strategic level of the health system. It also allows us to reflect on the role municipal management plays in influencing the health care model, its implications for health practices and for the reproduction of social health needs ^17^ ^,^ ^18^, which assumes renewed interest in the current scenario.

Given the complexity inherent to the discussions about PHC services organization, within the perspective of public policies and care models, as well as the relevance of analyzing health practices and their management, studies focusing on the interfaces between municipal management and the organization of work processes can help examine the incorporation of SUS ethical-political guidelines for PHC and its practices.

In this perspective, the São Paulo experience is a rich scenario for the proposed discussion given the diversity of PHC arrangements and their different response capacities ^19^ ^,^ ^20^ ^,^ ^21^, and the accumulated debate on health care models and public policy propositions - historically inspired by state experiences and critical reflections on PHC ^22^.

Given this panorama, the following question arises: “What are the different forms of basic network organization that can be identified in São Paulo municipalities?”, “Which of these organizations remain permeable to FHS guidelines?”, “Do indicators that reflect interfaces between municipal management and local management of services distinguish the basic network organizational models?”.

Hence, this study analyzes the main organization patterns of the São Paulo basic health care networks and evaluate them according to indicators of local management-administration interface based on the presence of components necessary for a comprehensive health care model.

## Methods

An evaluative, quantitative and cross-sectional research was conducted in the São Paulo municipalities that participated in the 2017 *Primary Care Services Quality Assessment Survey* (QualiAB), categorized according to the PHC organizational arrangements that make up their basic network.

Identifying the organizational patterns of municipal primary health care networks based on data collected by the QualiAB was possible because it is a validated instrument ^23^ ^,^ ^24^ that includes different types of services in the evaluation process according to their self-classification. Local managers or person responsible for coordinating the unit answer the QualiAB questionnaire in dialogue with the team, based on voluntary adherence by municipalities and units. It allows us to formulate indicators related to both the organization of health care actions and local management ^24^. Moreover, it has good adherence by managers from various regions in the State of São Paulo and has already demonstrated a high evaluative power, based on different scopes ^25^ ^,^ ^26^ ^,^ ^27^ ^,^ ^28^ ^,^ ^29^ ^,^ ^30^.

To define municipal standards, we aggregated the organizational arrangements self-identified by the units into three types: family health units - FHU; traditional basic health units - BHU (units without a family health team and without community health workers - CHW -, integrated or not into the emergency care unit, and outposts with mobile teams); and BHU/FHU (units self-defined as BHU with a CHW program and/or family health teams; and units self-defined as USF integrated with an emergency care unit). We used this classification to identify the municipal PHC organization patterns according to the types of services provided by the basic network of each municipality.

Our study included 2,472 services from 461 municipalities out of the 2,743 services located in 595 municipalities that participated in the survey. Inclusion criterion for municipalities consisted of the percentage of PHC services that responded to the QualiAB questionnaire (https://abasica.fmb.unesp.br/), varying according to the total number of units, to maintain the representativeness of the municipal network. Municipalities with more than six services were included if 75% or more of the total units answered the survey; municipalities with three to six services, 2/3 of the units; and municipalities with two services, if one service (50% of the municipality’s services) participated. Criteria variability according to the total number of services allowed us to include small municipalities while maintaining the simple majority criterion, since the 75% cut-off would exclude them.

Considering the key role played by municipal and local management interactions in the health care process, and guided by the SUS guidelines for PHC until 2017, we selected indicators related to local management whose occurrence requires guidance or authorization from the municipal management. We selected eight process indicators among those generated by the multiple-choice QualiAB questions to measure the influence of municipal management in unit organization. The selected indicators were grouped into three domains of analysis (planning, management and evaluation) detailed in Box 1.

**Box 1:** Local management indicators dependent on municipal health management, according to three domains. *Primary Care Services Quality Assessment Survey* (QualiAB), São Paulo, Brazil, 2017.

DOMAINS	INDICATORS
Planning	Definition of the catchment area by participatory planning considering the local reality.
	Use of care production data to guide and plan unit actions.
	Use of the unit’s care production data only by municipal management.
	Survey of the local reality over the last three years using data from programs, family registers, spontaneous demand and/or community studies.
Management	Weekly or fortnightly team meetings.
	Technical support by NASF and/or multiprofessional team from outside the unit’s staff.
Evaluation	Participation in evaluation processes organized by federal and/or state management.
	Unfolding of evaluations into the planning or reorganization of unit care strategies.

NASF: Family Health Support Centers.

Source: prepared by the authros.

To evaluate the municipal PHC network patterns that could identify the promotion of care models operationalized in PHC services, we investigated the association between the municipal typology and the selected indicators by means of distribution of frequencies of the variables, using chi-square tests with Bonferroni correction, followed by Z tests for proportions in statistically significant estimates (p ≤ 0.05). All analyses were performed using IBM SPSS v. 25.0 software (https://www.ibm.com/).

## Results

Patterns in the organization of municipal primary care networks were identified by recognizing two large groups within PHC services composition - municipalities with a “homogeneous” network, i.e., with all services of the same type; and municipalities with a “heterogeneous” network, i.e., made up of two or more types of PHC services. Internal division of these two groups resulted in seven subgroups according to PHC organizational arrangements (Box 2).

**Box 2:** Municipal primary care network patterns according to primary health care (PHC) services. *Primary Care Services Quality Assessment Survey* (QualiAB), São Paulo, Brazil, 2017.

BASIC HEALTH CARE NETWORK PATTERNS	BREAKDOWN BY TYPE OF SERVICE
Homogeneous: municipalities with PHC units with a single type of organizational arrangement	
1. Homogeneous-tradicional	Presence of BHU services only
2. Homogeneous-FHS	Presence of FHU services only
3. Homogeneous-mixed	Presence of BHU/FHU services only
Heterogenous: municipalities with PHC units with more than one type of organizational arrangement	
4. Heterogenous-FHS and mixed	Presence of FHU and BHU/FHU services
5. Heterogenous-traditional and mixed	Presence of traditional and BHU/FHU services
6. Heterogenous-FHS and traditional	Presence of FHU and traditional services
7. Heterogenous-FHS, traditional and mixed	Presence of FHU, traditional and BHU/FHU services

FHS: Family Health Strategy; BHU: basic health units; FHU: family health units.

Source: prepared by the authors.

Of the 461 municipalities evaluated, 201 (43.6%) were classified as homogeneous and 260 (56.4%) as heterogeneous (Table 1).

**Table 1:** Municipal primary care network patterns by distribution of the organizational arrangements of the services and according to the ratio of services per municipality. *Primary Care Services Quality Assessment Survey* (QualiAB), São Paulo, Brazil, 2017.

Municipal primary care network patterns	Services				Municipalities	Ratio of services per municipalities
	FHU	BHU/FHU	BHU	Total		
	n (%)	n (%)	n (%)	N (%)	N (%)	
Homogeneous	121 (10.6)	145 (22.5)	112 (16.2)	378 (15.3)	201 (43.6)	1.88
1. Homogeneous-traditional	0 (0.0)	0 (0.0)	112 (16.2)	112 (4.5)	50 (10.8)	2.24
2. Homogeneous-FHS	121 (10.6)	0 (0.0)	0 (0.0)	121 (4.9)	48 (10.4)	2.52
3. Homogeneous-mixed	0 (0.0)	145 (22.5)	0 (0.0)	145 (5.9)	103 (22.3)	1.41
Heterogeneous	1.017 (89.4)	499 (77.5)	578 (83.8)	2.094 (84.7)	260 (56.4)	8.05
4. Heterogeneous-FHS and mixed	274 (24.1)	187 (29.0)	0 (0.0)	461 (18.6)	71 (15.4)	6.49
5. Heterogeneous-traditional and mixed	0 (0.0)	60 (9.3)	44 (6.4)	104 (4.2)	22 (4.8)	4.73
6. Heterogeneous-FHS and traditional	292 (25.7)	0 (0.0)	197 (28.6)	489 (19.8)	74 (16.1)	6.61
7. Heterogeneous-FHS, traditional and mixed	451 (39.6)	252 (39.1)	337 (48.8)	1.040 (42.1)	93 (20.2)	11.18
Total	1,138 (100.0)	644 (100.0)	690 (100.0)	2.472 (100.0)	461 (100.0)	5.36

FHS: Family Health Strategy; BHU: basic health units; FHU: family health units.

Although most of the 2,472 services analyzed are characterized as FHU (n = 1,138), the 48 municipalities that only had services with this type of organizational arrangement (homogeneous-FHS) represent only 10.4% of the universe studied. Municipalities with a smaller number of services were concentrated on the homogeneous group, with the homogeneous-mixed subcategory standing out for covering the largest number of municipalities (n = 103) and having the lowest ratio of services per municipality (1.41) (Table 1).

The heterogeneous group included municipalities with the highest number of services, with a ratio of 8.05 services per municipality, with the heterogeneous-FHS, traditional and mixed subgroup standing out with a 11.18 ratio. Note that this subgroup also had the largest number of FHU, which together with the presence of the heterogeneous-FHS and traditional and heterogeneous-FHS and mixed subgroups accounted for 89.4% of the participating FHU (Table 1).

Table 1 also shows that the municipalities classified as homogeneous-mixed (22.3%) and heterogeneous-FHS, traditional and mixed (20.2%) were the most represented in the universe studied, highlighting the presence of BHU/FHU units, i.e., services organized in the traditional arrangement but which include FHS components, such as family health teams and/or CHW.

To better understand the municipality profile of these groups, we distributed the municipalities by population stratum according to municipal classification. This analysis showed that most municipalities with up to 10,000 inhabitants (n = 203) are homogeneous (72.4%), with BHU/FHU units (44.3%). In municipalities with more than 10,000 inhabitants, the heterogeneous arrangement gains importance as the population size increases (Table 2).

**Table 2:** Municipal primary care network patterns according to population strata. *Primary Care Services Quality Assessment Survey* (QualiAB), São Paulo, Brazil, 2017.

Municipal primary care network patterns	Population strata (inhabitants)			Total
	Up to 10,000	10-50,000	> 50,000	
	n (%)	n (%)	n (%)	N (%)
Homogeneous	147 (72.4)	45 (26.2)	9 (10.5)	201 (43.6)
1. Homogeneous-traditional	30 (14.8)	13 (7.6)	7 (8.1)	50 (10.8)
2. Homogeneous-FHS	27 (13.3)	21 (12.2)	0 (0.0)	48 (10.4)
3. Homogeneous-mixed	90 (44.3)	11 (6.4)	2 (2.3)	103 (22.3)
Heterogeneous	56 (27.6)	127 (73.8)	77 (89.5)	260 (56.4)
4. Heterogeneous-FHS and mixed	15 (7.4)	44 (25.6)	12 (14.0)	71 (15.4)
5. Heterogeneous-traditional and mixed	7 (3.4)	11 (6.4)	4 (4.7)	22 (4.8)
6. Heterogeneous-FHS and traditional	26 (12.8)	29 (16.9)	19 (22.1)	74 (16.1)
7. Heterogeneous-FHS, traditional and mixed	8 (3.9)	43 (25.0)	42 (48.8)	93 (20.2)
Total	203 (100.0)	172 (100.0)	86 (100.0)	461 (100.0)

FHS: Family Health Strategy.

Source: Brazilian Institute of Geography and Statistics ^43^.

Of the 172 municipalities with a population between 10,000 and 50,000 inhabitants, 73.8% were concentrated in the heterogeneous typology, especially in the heterogeneous-FHS and mixed and heterogeneous-FHS, traditional and mixed groups (25.6% and 25%, respectively) (Table 2).

Among those with more than 50,000 inhabitants, 89.5% have heterogeneous basic networks, made up mainly of municipalities with a heterogeneous-FHS, traditional and mixed and heterogeneous-FHS and traditional pattern (48.8% and 22.1%, respectively). In this group of 77 larger municipalities, only nine were classified as homogeneous, seven of them with traditional services and two with BHU/FHU units (Table 2).

Associations between the seven groups of municipalities, classified according to basic network composition, and the selected indicators showed significant differences between them (p < 0.001) (Table 3).

**Table 3:** Distribution of municipalities with homogeneous and heterogeneous patterns according to selected management indicators by domain. *Primary Care Services Quality Assessment Survey* (QualiAB), São Paulo, Brazil, 2017.

	Homogeneous municipalities			Heterogenous municipalities				p-value **
	Traditional (n = 112)	FHS (n = 121)	Mixed * (n = 145)	FHS + Mixed * (n = 461)	Traditional + Mixed * (n = 104)	FHS + Traditional (n = 489)	FHS + Mixed * + Traditional (n = 1,040)	
	%	%	%	%	%	%	%	
Planning								
Definition of the catchment area by participatory planning considering the local reality	21 (18.8)^a^	48 (39.7)^b^	64 (44.1)^b^	111 (24.1)^a^	17 (16.3)^a^	94 (19.2)^a^	243 (23.4)^a^	< 0.001
Use of the unit’s care production data only by municipal management	63 (56.3)^a^	32 (26.4)^b^	43 (29.7)^b^	146 (31.7)^b^	57 (54.8)^a^	201 (41.1)^a,b^	358 (34.4)^b^	< 0.001
Use of care production data to guide and plan unit actions	44 (39.3)^a^	85 (70.2)^b^	96 (66.2)^b,c^	299 (64.9)^b^	43 (41.3)^a^	255 (52.1)^a,c^	637 (61.3)^b^	< 0.001
Survey of the local reality over the last three years using data from programs, family registers, spontaneous demand and/or community studies	55 (49.1)^a^	108 (89.3)^b^	126 (86.9)^b,c^	406 (88.1)^b^	85 (81.7)^b,c,d^	364 (74.4)^d^	790 (76.0)^c,d^	< 0.001
Management								
Weekly or fortnightly team meetings in the last year	25 (22.3)^a^	81 (66.9)^b^	53 (36.6)^a,c^	286 (62.0)^b^	35 (33.7)^a,c^	252 (51.5)^d^	472 (45.4)^c,d^	< 0.001
Technical support by NASF and/or multiprofessional team from outside the unit’s staff	51 (45.5)^a^	85 (70.2)^b.c^	95 (65.5)^c^	386 (83.7)^d^	81 (77.9)^b,c,d^	355 (72.6)^c^	832 (80.0)^b,d^	< 0.001
Evaluation								
Participation in evaluation processes organized by federal and/or state management in the last three years	27 (24.1)^a^	104 (86.0)^b,c^	135 (93.1)^c^	374 (81.1)^b^	54 (51.9)^d^	317 (64.8)^d^	669 (64.3)^d^	< 0.001
Unfolding of evaluations into the planning or reorganization of care strategies	28 (25.0)^a^	88 (72.7)^b,c^	113 (77.9)^c^	326 (70.7)^c^	52 (50.0)^d^	287 (58.7)^b,d^	635 (61.1)^b,d^	< 0.001

FHS: Family Health Strategy; NASF: Family Health Support Centers.

* Mixed: presence of basic health units (BHU)/family health units (FHU);

** Chi-square test with Bonferroni correction followed by Z test when p-value < 0.05.

Note: values followed by the same letter indicate no statistically significant difference between percentages.

When considering the Z-test correction, homogeneous-traditional and homogeneous-FHS stand out with significant differences in all the indicators. On the other hand, municipalities whose basic network composition classifies them as homogeneous-mixed and heterogeneous-FHS/mixed have percentages similar to homogeneous-FHS in almost all indicators. Municipalities with networks that include the different types of services considered - heterogeneous FHS, traditional and mixed - have indicators that are sometimes closer to those with FHS and sometimes closer to those with arrangements that include traditional services (Table 3).

In general, the municipalities classified as homogeneous-traditional had the lowest concentration of services with external technical support, least participated in evaluation processes organized by federal and/or state management and least used their results to plan or reorganize care strategies. They also had the lowest concentration of services that collected information on the local reality (Table 3).

Among the planning indicators, “definition of the catchment area by participatory planning, considering the local reality” is the basis for the territorialization process. However, the highest frequency occurred only in the homogeneous-FHS and mixed networks (39.7% and 44.1%), which differentiated them from the others, since all those with a traditional component were close to the lowest values of this indicator (Table 3).

The “use of care production data only by municipal management” indicator is also closer to municipalities with a traditional component, while “use of care production data to guide and plan unit actions”, with a focus on local planning, is more frequent and does not differentiate between the homogeneous-FHS and mixed and the heterogeneous-FHS/mixed groups. Similar behavior occurs in relation to survey of the local reality (Table 3).

We highlight the groups that concentrated a greater number of services with weekly or fortnightly team meetings, a practice employed by most of the services located in the municipalities of the homogeneous-FHS and heterogeneous-FHS and mixed groups (66.9% and 62%, respectively). Regarding this indicator, only 36.6% of the services in the homogeneous-mixed group reported weekly or fortnightly team meetings, which resembles what happens in groups with traditional components (Table 3).

On the other hand, “technical support by Family Health Support Centers (NASF) and/or a multiprofessional team from outside the unit” was frequent in all the different municipal network compositions, excepting homogeneous-traditional.

Indeed, municipalities classified as homogeneous-FHS and homogeneous-mixed showed similar results to the heterogeneous-FHS and mixed municipalities in most of the indicators, especially those that show compliance with FHS recommendations, such as concentrating municipalities with a greater number of PHC services that declared having participated in the evaluation processes organized by federal and/or state management and that used the results of previous evaluations to plan or reorganize care service strategies (Table 3).

## Discussion

We identified different PHC organization patterns in a significant number of São Paulo municipalities. Corroborating evidence of this diversity in other Brazilian regions ^1^ ^,^ ^31^, result analysis showed that the organizational arrangements of PHC services in São Paulo State were guided by FHS guidelines, even if not fully incorporated as a substitute model. Even amidst this great heterogeneity of organizational arrangements, the indicators point to actions that configure conditions for implementation of FHS-guided practices and, although they lack a direct association with health care actions, they indicate mechanisms that enable and strengthen them.

Despite the major presence of the FHS arrangement among the participating services, smaller municipalities, with one or two PHC services in their territory, have a tendency to implement mixed networks, with BHU/FHU units. Integration of family health teams and/or CHW into the organization of work processes increase the possibilities of reproducing the same response patterns operated according to the FHS model, which has already been noted by other evaluative studies and differentiate them from traditional units ^19^ ^,^ ^21^ ^,^ ^31^ ^,^ ^32^ ^,^ ^33^.

Our results confirm the large number of municipalities with heterogeneous networks such as heterogeneous-FHS, traditional and mixed, composed mostly of municipalities with more than 50,000 inhabitants, but which also includes a significant part of municipalities with 10,000 to 50,000 inhabitants. This high frequency of municipalities with the three types of services coincides with other studies on the coexistence of different PHC arrangements in São Paulo municipalities ^19^ ^,^ ^20^ ^,^ ^21^ ^,^ ^22^ and the resistance of some of them, especially those with more than 100,000 inhabitants, to fully adhering to the FHS ^33^ ^,^ ^34^.

Municipalities with FHS or mixed arrangements and a predominance of multiprofessional support provided by the NASF and/or external multiprofessional teams showed greater alignment with FHS guidelines, which can indicated, albeit indirect, interdisciplinary work aimed at comprehensive care ^1^ ^,^ ^2^. This result is all the more relevant given that the changes in PHC advocated by current policies represent a strong threat to care models that value comprehensive and equitable care provided by multiprofessional teams ^12^.

In this regard, we highlight the municipalities that make up the homogeneous-FHS, homogeneous-mixed and heterogeneous-FHS and mixed groups and which therefore have family health teams and/or CHW, whose indicators show higher frequencies in the use of care production data in the planning of actions offered by the unit and in the collection of data to understand the local reality, which suggests different investments regarding community commitment in the organization of actions conducted by these services ^35^.

Municipalities belonging to the homogeneous-FHS and mixed groups showed greater definition of the catchment area by participatory planning considering the local reality, which can be pointed out as another important characteristic related to the type of territorialization and client registration, a fundamental attribute in implementing the care model advocated by the FHS. Notably, although the need to plan according to local reality has been imposed in the formulation of community actions and comprehensive care, its implementation has always remained a challenge for the SUS; however, as in other countries, it is under greater threat of the fiscal austerity measures introduced in response to the economic crisis ^3^ ^,^ ^5^ ^,^ ^6^ ^,^ ^7^ ^,^ ^10^ ^,^ ^36^.

The greater participation in the evaluation processes observed in the three groups of municipalities with FHS components and no traditional services must be interpreted with caution. Although it may indicate greater involvement and/or understanding of the benefits of these processes in improving organizational quality, it may also reflect the influence of evaluation models implemented by the Federal Government - which until 2014 only allowed the participation of services organized according to the FHS model ^37^. On the other hand, seeing as these municipalities had the highest number of services claiming incorporation of the results of evaluation processes into unit action planning, we can reaffirm the value placed on evaluation processes for incorporating changes into work processes and, consequently, improving the quality of care provided ^37^ ^,^ ^38^.

The three groups with the most FHS components indicators - Homogeneous-FHS, homogeneous-mixed and heterogeneous-FHS and mixed - showed greater frequency of weekly or fortnightly team meetings. This is a prerequisite for teamwork, with the ability to directly influence care coordination and enable participatory management. While the meeting space can indeed be absorbed by bureaucratic and organizational issues, it is also the space where planning and case discussion takes place ^39^ to enable teamwork.

Despite the great heterogeneity of organizational arrangements, as evidenced in PHC services in other Brazilian states ^31^, the municipalities with homogeneous-FHS, homogeneous-mixed and heterogeneous-FHS and mixed stood out positively in relation to most indicators as management constraints for a comprehensive and integral PHC model. On the other hand, the homogeneous-traditional group, whose services does not include relevant FHS elements, stands out negatively in relation to the same variables.

Such a scenario points to an alignment with the FHS model in the period studied, even within the conformation of municipalities with BHU/FHU-type services. This trend highlights the strategic position of municipal managers in implementing policies that adapt their practices to the local reality, as it shows that the organizational, planning and management measures taken are important in enabling work processes with greater capacity to respond to the health needs of each territory. It also highlights the inductive power of federal and state policies in the incorporation of this model ^40^ ^,^ ^41^ by municipal administrations. 

Considering the relative autonomy given to municipal managers in the regulations currently in force regarding PHC management and financing - which allow municipalities to adapt the recommendations ^14^ ^,^ ^15^ ^,^ ^42^ - highlights the strategic role of monitoring and evaluation processes for identifying and following up on the possible impacts of counter-reform policies.

These results become worrying in a context where federal management regulations issued in recent years for this level of care, such as the 2017 Brazilian National Primary Helth Care Policy (PNAB) and the Previne Brasil program, represent proposals that open up space to mischaracterize team interdisciplinarity, to bureaucratize the role of CHW and to reduce health promotion actions, thus hindering the consolidation of FHS-based care. But, above all, by pushing back the consolidation of a strong PHC and a public and universal SUS ^5^ ^,^ ^6^ ^,^ ^7^ ^,^ ^8^ ^,^ ^10^ ^,^ ^11^.

As for study limitations, we can cite the use of indicators which, although they proved to be sensitive to the associations investigated, did not allow for a closer identification of the care model implemented by the services. Moreover, although the universe analyzed includes a significant number of municipalities and PHC services, it refers to a statewide sample of voluntary adherence, answered by the unit manager and, therefore, ignoring the perspective of administrators for this network configuration.

However, this analysis proved to be valid and replicable in other contexts, providing a better understanding of municipal primary care networks, which includes FHS organization and the type of family health teams and/or CHW incorporation. It also allowed us to identify elements for debating existing weaknesses and alternatives for strengthening the process of incorporating FHS guidelines.

## Final considerations

Our results point to a significant presence of the FHS under the previous PNAB - which clearly prioritized this model of health care organization, including allocations for its expansion. Even in a scenario where different service organization models coexist, the municipal health care networks with family health teams and/or CHW presented general characteristics closer to those recommended by the SUS guidelines for PHC, reinforcing the power federal and state managements have to induce changes in the care model, and the strategic position municipal managers play in implementing policies that affect care practices organization.

The varied organizational arrangements identified seems to mirror the historical construction of PHC in São Paulo, but also suggests the political-institutional choices made by municipal managers, which may be associated with professional resistance to adhering to changes in traditional health care models.

Our findings also suggest that raising awareness about FHS incorporation in managers tends to result in a services organization more porous to incentives and debates about comprehensiveness, advancing SUS consolidation. Managers must avoid leaving the heterogeneity of existing arrangements in São Paulo to be exploited in the name of reducing FHS practices and, at the same time, should strengthen measures that promote the incorporation of FHS guidelines in PHC services organization.

By shedding light on strategic elements for PHC strengthening we expect to raise the three levels of management awareness to the importance of including family health teams and CHW in services (re)organization according to the guidelines of a comprehensive PHC, with universal and equitable access, thus strengthening municipal commitments to quality care in the SUS.
